# Successful percutaneous coronary intervention of left main coronary artery dissection following mechanical aortic valve replacement surgery: a case report and literature review

**DOI:** 10.3389/fcvm.2024.1451194

**Published:** 2024-08-21

**Authors:** Vu Hoang Vu, Hung Phi Truong, Hoa Tran, Khang Dang Cao, Bao Thien Duong, Thuy Thanh Thi Tran, Binh Quang Truong

**Affiliations:** ^1^ Medicine Faculty, University of Medicine and Pharmacology at Ho Chi Minh City, Ho Chi Minh City, Vietnam; ^2^ Interventional Cardiology Department, University Medical Center Ho Chi Minh City, Ho Chi Minh City, Vietnam; ^3^ Pediatric Cardiovascular Surgery Department, University Medical Center Ho Chi Minh City, Ho Chi Minh City, Vietnam; ^4^ Cardiac Intensive Care Unit, University Medical Center Ho Chi Minh City, Ho Chi Minh City, Vietnam

**Keywords:** iatrogenic left main coronary artery dissection, mechanical aortic valve replacement surgery, coronary artery bypass surgery, percutaneous coronary intervention, intravascular ultrasound

## Abstract

**Background:**

Iatrogenic left main coronary artery (LMCA) dissection resulting from cardiac surgery is a rare complication. Its early detection is challenging and often poses a significant threat to the patient's life. However, evidence regarding the most effective management strategy for this condition remains limited at present.

**Case presentation:**

We present a case of 65-year-old female patient who developed cardiogenic shock after mechanical aortic valve replacement surgery associated acute myocardial infraction. Despite concurrent coronary artery bypass graft (CABG) surgery, the patient's condition remained unimproved. Subsequent coronary angiography revealed extensive LMCA dissection involving the left circumflex (LCx) artery. Percutaneous coronary intervention (PCI) guided by intravascular ultrasound (IVUS) led to an immediate improvement in hemodynamic status. The patient was successfully discharged after 22 days of treatment.

**Conclusions:**

Iatrogenic LMCA dissection is an uncommon complication following cardiac surgery. It can manifest in a variety of ways, including as incidental findings, cardiogenic shock or sudden cardiac arrest. The precise prevalence rates of causes linked to cardiac surgery remain largely unknown due to the scarcity of reported cases and the absence of research on this issue. Currently, a definitive management strategy for this condition has not been established. However, previous reported clinical cases provide insight that CABG could be considered if coronary artery dissection is detected during cardiac surgery. Upon postoperative identification, diagnostic coronary angiography and PCI may be feasible alternatives.

## Introduction

Iatrogenic LMCA dissection is a rare occurrence that arises from the manipulation of a guidewire or catheter during coronary angiography or interventional procedures. The incidence rate of this event is less than 0.1% (1). LMCA dissection resulting from cardiac surgery is extremely uncommon, with diverse clinical presentations ranging from incidental detection to life-threatening arrhythmias, hemodynamic instability, or progression to cardiogenic shock.

In the present case, a 65-year-old female underwent redo aortic valve surgery and subsequently developed cardiogenic shock secondary to acute myocardial infarction. Despite undergoing concurrent CABG surgery, the patient still experienced severe metabolic acidosis and life-threatening ventricular arrythmias. Subsequent coronary angiography revealed extensive LMCA dissection. A PCI was appropriately performed with the assistance of IVUS, leading to an improvement in the patient's hemodynamic condition. Additionally, a literature review was conducted on this condition. A search on PubMed using the keywords “dissection”, “left main coronary artery”, and “aortic valve replacement surgery” yielded 37 results, from which 5 case reports were included in the final review. Exclusion criteria included duplication, absence of specific dissection reports, and unavailability of full texts.

## Case presentation

**Figure 1:**
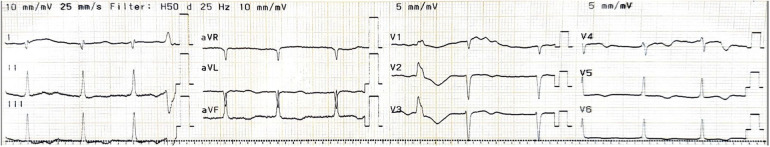
Pre-operative electrocardiogram. Atrial fibrillation with moderate ventricular response accompanied by T wave inversion in V2-6, poor R wave progression and premature ventricular complex.

A 65-year-old female patient presented to the hospital with a one-month history of exertional dyspnea. Her medical history included permanent atrial fibrillation and mechanical mitral and aortic valve replacement, along with the placement of a bioprosthetic tricuspid valve ring 20 years prior, due to rheumatic heart disease. The patient has no prior history of genetic disorders or neuropsychiatric conditions. Furthermore, there is no documented family history of genetic disorders. She was on daily medication consisting of acenocoumarol 1 mg once daily and bisoprolol 2.5 mg once daily. Upon admission, physical examination revealed stable vital signs, with a grade 4/6 systolic murmur audible along the left sternal border. Laboratory tests indicated normal renal function, with a creatinine level of 0.55 mg/dl (normal range: 0.66–1.09 mg/dl), and a slightly elevated NT-proBNP concentration of 322 ng/L (normal range: <125 ng/L). (Figure 1) illustrates the patient's electrocardiogram (ECG) findings at the time of admission.

Echocardiography findings indicated normal left and right ventricular function as well as proper positioning and function of the bioprosthetic tricuspid and mechanical mitral valve. However, the mechanical aortic valve was severely stenosed, with a mean pressure gradient of 52 mmHg and a maximal velocity of 4.88 m/s across the valve. Additionally, a chest computed tomography (CT) scan revealed restricted aortic valve opening motion in the absence of a substantial thrombus. The assessment of the coronary artery system also revealed no significant stenosis.

The patient was diagnosed with symptomatic severe restenosis of mechanical aortic valve and underwent valve replacement surgery. The duration of the operation was ten hours and thirty minutes. Initially, extracorporeal circulation (with cardiopulmonary bypass) was established, and cardioplegia infusion was administered. The restricted movement of the leaflets of the mechanical aortic valve, when the ascending aorta was opened, was attributed to panus. The mechanical aortic valve was subsequently substituted with a size 21 On-X valve. Ventricular fibrillation occurred after the left ventricle was de-aired and the aortic clamp was released; therefore, initial biphasic defibrillation with 20J twice and 30J once was performed. Subsequent monitoring revealed atrial fibrillation with rapid ventricular response. Transesophageal echocardiography confirmed adequate left ventricular contractility, proper function of the mechanical aortic valve, and intact blood flow into the left anterior descending artery (LAD), LCx, LMCA while the patient was gradually weaned off bypass.

Nevertheless, recurrent ventricular fibrillation was observed during suturing, which persisted despite two ventricular defibrillation attempts. After re-establishing extracorporeal circulation and exposing the ascending aorta, it was observed that the RCA was patent, while the left coronary arteries was partially occluded by the mechanical aortic valve. As a result, a CABG was performed, utilizing a right great saphenous vein to provide two bypasses from the aorta to the LAD and RCA. Despite being administered four different vasopressors and inotropes (milrinone, dobutamine, adrenaline, and noradrenaline), the patient's mean arterial blood pressure remained inadequate, fluctuating between 55 and 60 mmHg. Afterwards, hemodynamic support with a counterpulsation balloon in the descending aorta was initiated.

The patient's hemodynamic status gradually deleriorated on the initial postoperative day despite the escalation in dosages of vasopressors and inotropes. Ventricular tachycardia was consistently observed on the monitor. Laboratory tests revealed profound metabolic acidosis with increasing blood lactate levels and a surge in troponin T levels from 4,168 to 5,803 ng/L, eventually exceeding 10,000 ng/L. Bedside echocardiography indicated a decrease in left ventricle ejection fraction (LVEF) from 53.8% to 36% and a reduction in mean aortic valve pressure gradient from 52 to 21 mmHg. NT-proBNP levels rose from 322 to 5,461 pg/ml. Additionally, there was modest renal impairment, evidenced by an increase in serum creatinine from 0.55 to 1.27 mg/dl, while hemoglobin levels remained relatively stable (130–133 g/L). The established diagnosis at this point was cardiogenic shock secondary to a very high-risk non-ST elevation myocardial infarction, following postoperative redo mechanical aortic valve and CABG.

**Figure 2:**
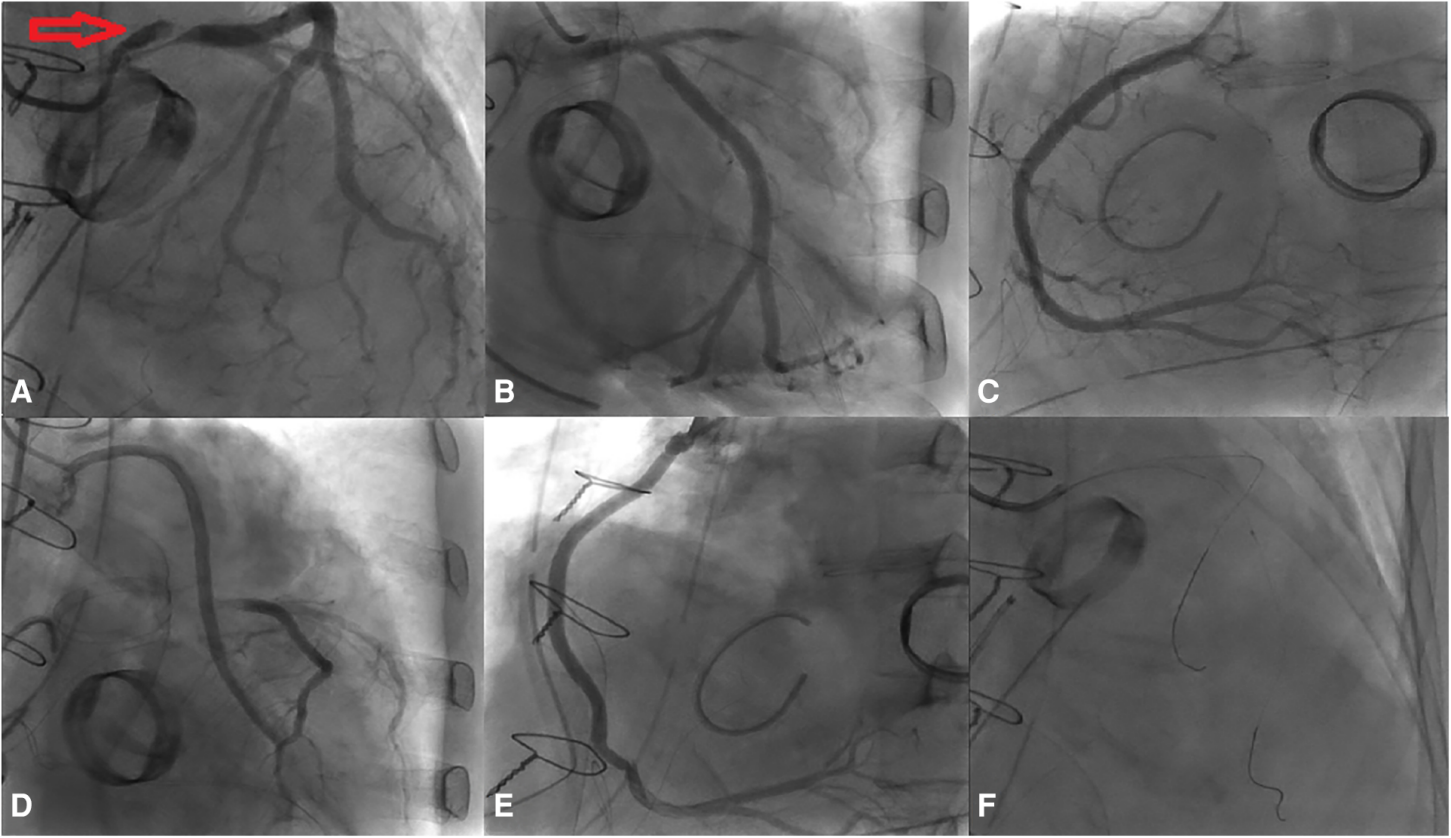
Results of the coronary angiography. **(A)** Dissection extending from the LM to the LCx (indicated by red arrow). **(B–E)** No significant stenosis observed in the LAD, RCA, Ao-vein-LAD, and Ao-vein-RCA. **(F)** Passage of the wire through both the LAD and the LCx, accompanied by an IVUS assessment.

Coronary angiography was subsequently performed on the patient via the femoral artery approach. The result demonstrated severe stenosis of the LMCA, with dissection extending into the ostium of the LCx. No significant stenosis was observed in the LAD, RCA, or bypass grafts. Using a sion wire, the lesion in the distal LCx was successfully crossed, after which a sion blue wire was advanced towards the distal LAD (Figure 2).

**Figure 3:**
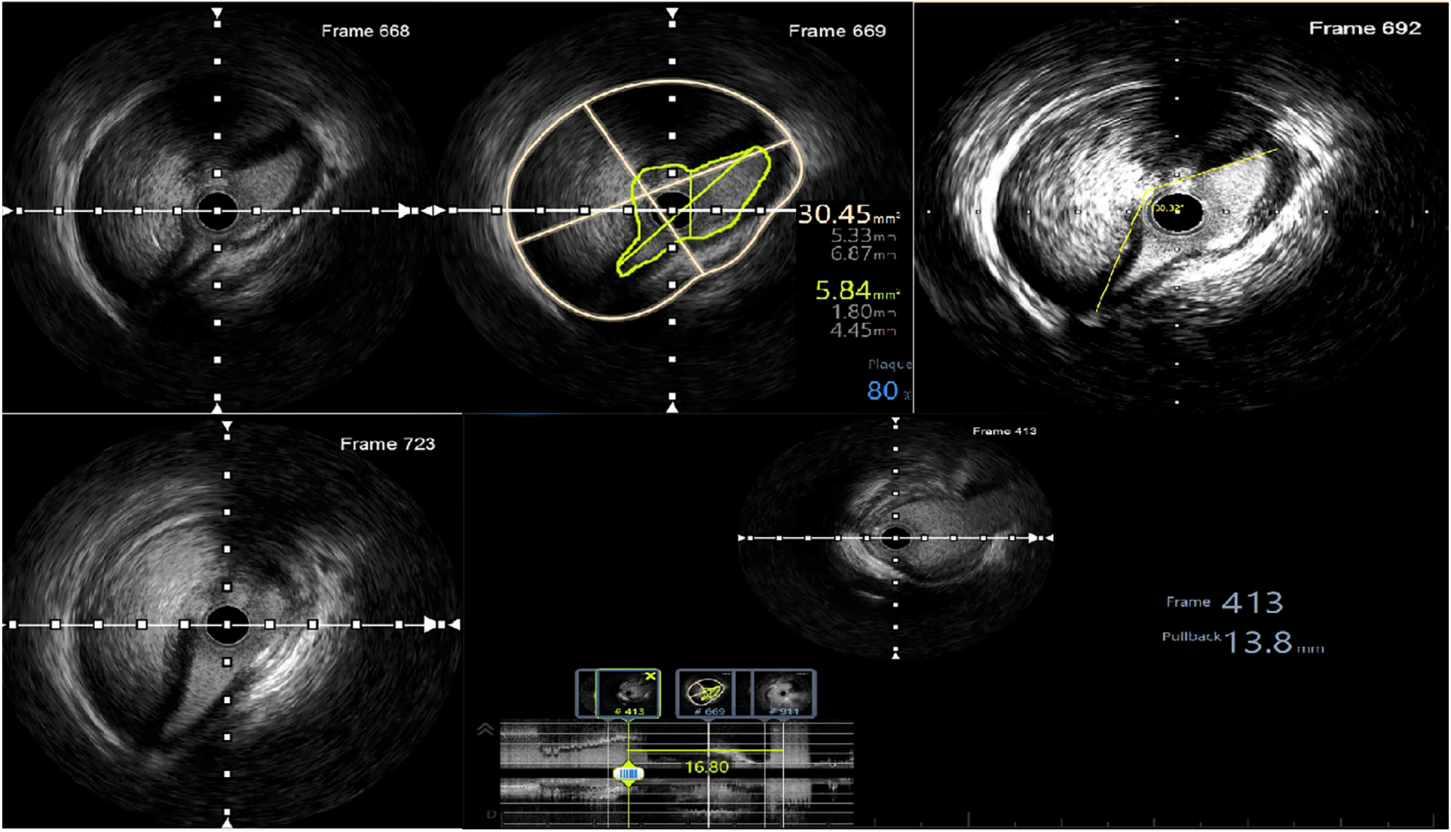
IVUS images of the LMCA dissection.

Afterwards, IVUS was used to assess the lesion. The findings indicated an absence of dissection in the LAD. However, examination of the LMCA to the LCx showed images suggesting complete separation of the intima-media from the adventitia, along with the presence of intramural hematoma. The initial dissection site extended approximately 16.8 mm from the ostium of the LMCA to the ostium of the LCx. The smallest true lumen area was measured at 5.84 mm^2^, resulting in an 81% stenosis compared to the vessel lumen area of 30.45 mm^2^. The dissection angle measured at this site was 230 degrees (Figure 3). The distal reference diameter of the landing zone was determined to be 4 mm (ranging from 3.85 to 4.14 mm), with a plaque burden of 22%.

**Figure 4:**
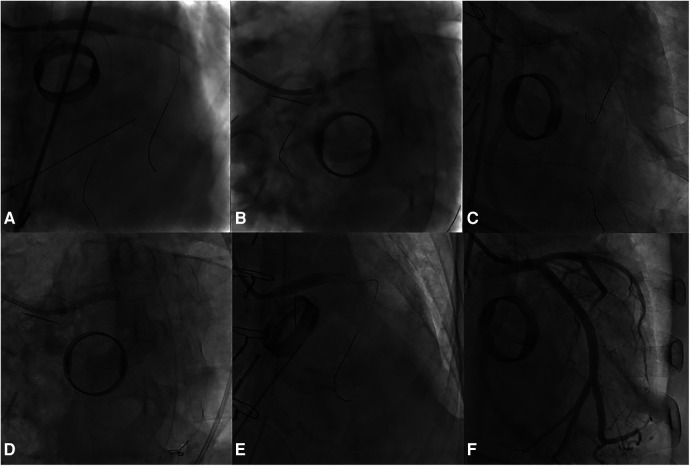
The PCI procedure for the LMCA and the LCx. **(A)** Deployment of a DES in the LMCA-LCx. **(B)** Initial Proximal Optimization Technique (POT) in the LM using a NC balloon. **(C)** Subsequent rewiring and dilation of the LAD ostium with a NC balloon. **(D)** In-stent post-dilation. **(E)** Second POT in the LMCA. **(F)** Final angiographic result.

A 4.0 × 24 mm drug-eluting stent (DES) was deployed in the LMCA-LCx, followed by post-dilation of the LMCA stent segment using a non-compliant (NC) balloon measuring at 5.0 × 15 mm. Subsequently, the sion blue wire was re-wired to the distal LAD, and the ostium of LAD was dilated with a 3.0 × 20 mm NC balloon. Following the initial post-dilation within the stent with a 4.0 × 20 mm NC balloon, second post-dilation was performed on the LMCA stent segment using a 5.0 × 15 mm NC balloon. Post-procedure coronary angiography confirmed TIMI 3 flow in the LMCA and LCx (Figure 4).

**Figure 5:**
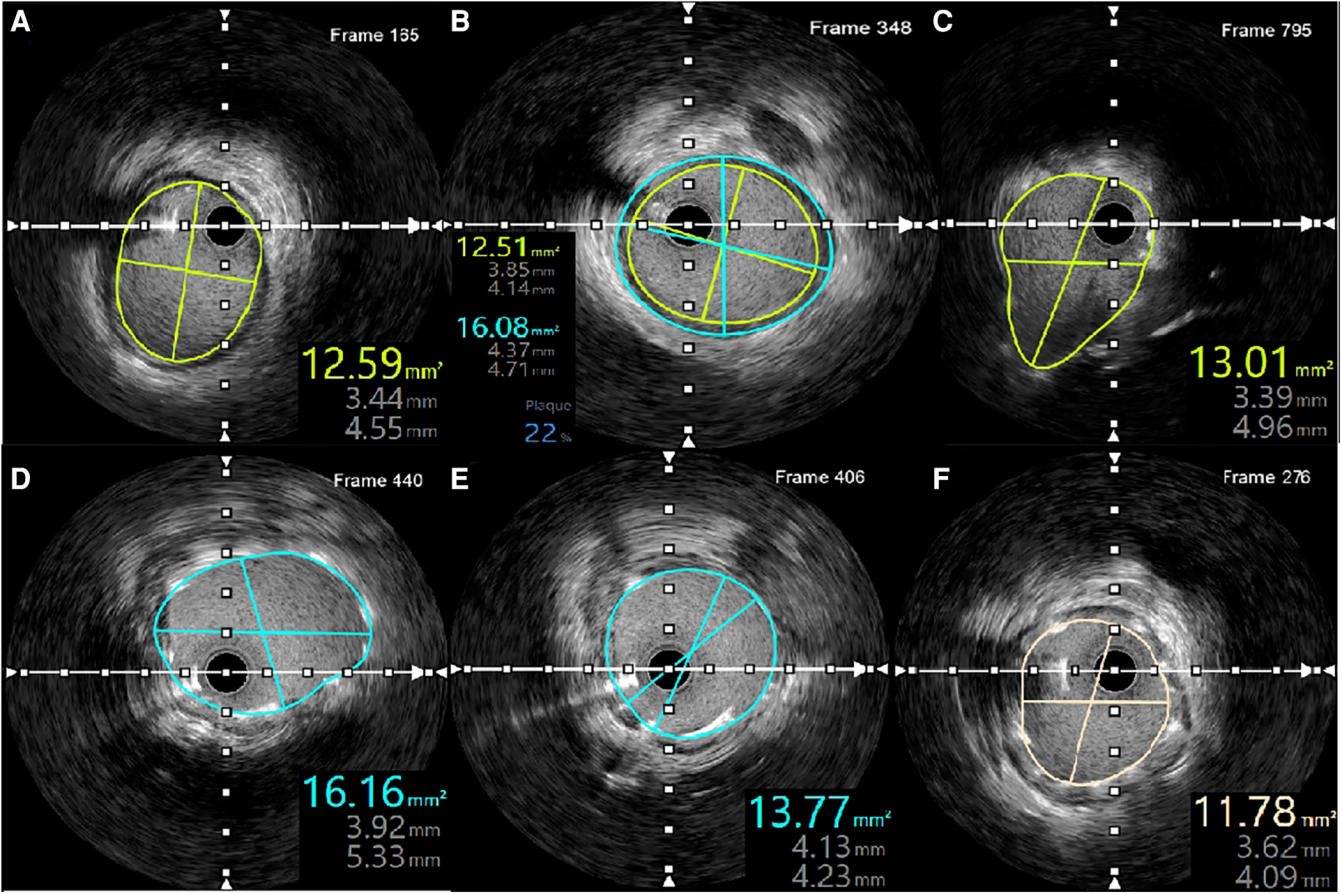
IVUS measurements following LMCA-LCx PCI. **(A)** MSA of the distal reference. **(B)** Plaque burden and distal reference diameter. **(C)** MSA at the proximal LMCA. **(D)** MSA at the distal LMCA. **(E)** MSA at the ostium of the LCx. **(F)** Smallest MSA within the stent.

IVUS revealed no evidence of dissection at either the proximal or distal stent edges, as well as the absence of tissue prolapse following PCI. The minimal stent area (MSA) at the distal and proximal ends of the LMCA were measured at 16.16 mm^2^ and 13.01 mm^2^, respectively. Meanwhile, the MSA at the LCx ostium was 13.77 mm^2^. The smallest MSA within the stent was 11.78 mm^2^, accounting for 94% of the MSA at the distal reference, which was 12.59 mm^2^. Additionally, IVUS verified adequate stent apposition at both proximal and distal ends (Figure 5).

**Figure 6:**
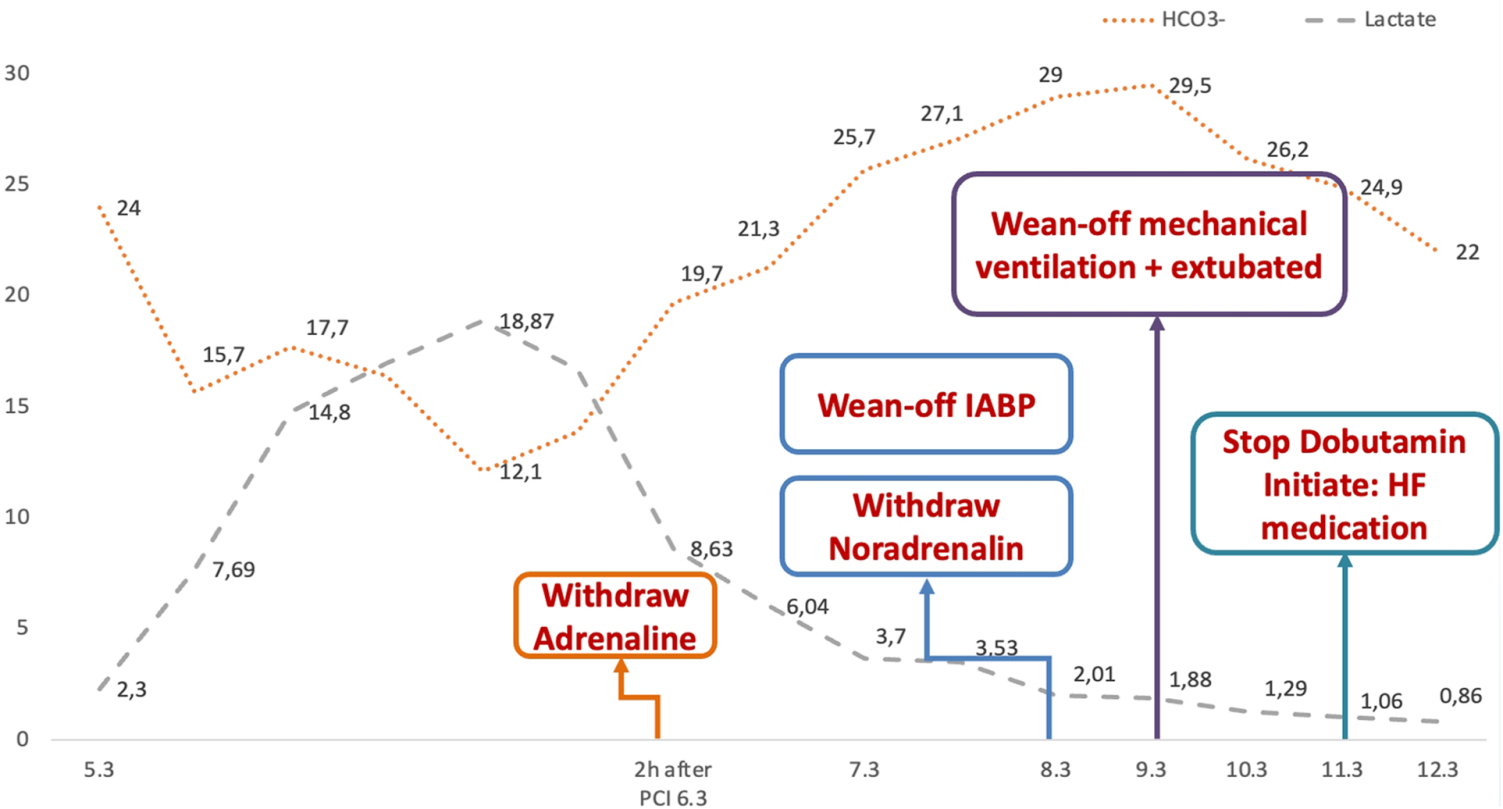
Dynamics of blood lactate and bicarbonate (HCO3-) levels during hospitalization.

The patient's hemodynamics status and metabolic acidosis condition showed significant improvement post-procedure (Figure 6). Vasopressors and inotropes was gradually tapered and ceased entirely after 5 days following PCI. In addition, the patient was successfully weaned off mechanical ventilation and extubated. Laboratory tests revealed a decline in NT-proBNP levels, decreasing from 5,461 to 1,501 ng/L. A further echocardiography performed 14 days later revealed an enhancement in LVEF, rising from 36% to 51.7%. The patient was discharged after 22 days in a stable condition and was prescribed the following medications: warfarin, clopidogrel, spironolactone, dapagliflozin, atorvastatin, and valsartan/sacubitril. The patient continued to be monitored at the outpatient clinic and remained in a stable condition. An echocardiography performed two months after discharge indicated that the mechanical mitral and aortic valves were functioning well, with the mean pressure gradient across the aortic valve reduced from 52 mmHg to 8 mmHg and the maximum velocity decreased from 4.88 m/s to 2.61 m/s. However, the LVEF remained low at 38%. Laboratory tests revealed that the NT-proBNP level had decreased to 746 ng/L, and the INR fluctuated significantly between 1.7 and 2.75. Over the past four months, the patient attended four follow-up visits, with average heart rate and blood pressure readings of 80 beats per minute and 130/80 mmHg, respectively. The patient's treatment regimen remained relatively constant during these visits, including warfarin 3 mg, clopidogrel 75 mg, valsartan/sacubitril (total dose 100 mg), spironolactone 25 mg, furosemide 10 mg, and bisoprolol 1.25 mg.

## Discussion

Iatrogenic coronary dissection following cardiac surgery has been occasionally reported. Although the incidence of this complication is not well-documented, early detection remains challenging and often results in extensive myocardial infarction. Management options include PCI, CABG or conservative therapy. However, there is limited evidence regarding the optimal approach for managing iatrogenic LMCA dissection complicating cardiac surgery (2).

Most complications arising from interventional procedures are typically identified promptly, either through ischemic symptoms or angiographic signs of dissection. However, in anesthetized patients undergoing cardiac surgery, symptoms and signs of ischemia may not be apparent if coronary flow remains unimpeded. Berroya et al. documented three cases of fatal LMCA dissection,in which all three patients unfortunately died and underwent postmortem evaluation (3). Conversely, Nakao et al. accidentally detected LMCA dissection using intraoperative transesophageal echocardiography, highlighting its potential diagnostic utility (4). Yukang et al. similarly reported a case with a clinical context resembling ours, where the complication was only recognized following myocardial infarction-induced hemodynamic instability. LMCA dissection exhibits diverse clinical manifestations, ranging from incidental detection to symptoms such as low cardiac output syndrome or cardiogenic shock, and even sudden death. Therefore, it is crucial to closely monitor vital signs, ECG, transthoracic echocardiography, and changes in cardiac injury markers postoperatively to facilitate early detection and develop appropriate treatment strategies. (Table 1) outlines the occurrences of coronary artery dissection subsequent to valve replacement surgery, as documented up to date.

**Table 1:** Summary of clinical cases reporting coronary artery dissection following cardiac surgery.

Author	Year of publication	Age	Gender	Type of surgery	Diagnosis method	Type of intervention	Clinical prensentation	Outcomes
Berroya et al. (3)	1970	Unknown	Unknown	Aortic valve replacement	Postmotem evaluation	None	Sudden cardiac arrest	Death
Nakao et al. (4)	2017	73	Male	Aortic valve replacement	Accidentally via transesophageal echocardiography identified intimal flap within LMCA lumen	OCT guidance PCI	Accidentally	Survival
Molek et al. (5)	2019	Case series: 3 patients with average age: 73 (minimum: 56–maximum: 85)	2 female 1 male	Aortic valve replacement	Coronary angiography identified LMCA dissection	PCI	Acute MI following the surgery	2 survival 1 death
Yu Kang et al. (2)	2020	43	Female	Aortic and mitral valve replacement	Coronary angiography identified a dissection from LMCA to LAD and LCx	None	Acute MI induced low-cardiac output syndrome	Survival
Haval Sadraddin et al. (6)	2021	73	Female	Aortic valve replacement	Coronary angiography identified diffuse coronary dissection from the ostium of the LAD to the left coronary system	CABG	Acute MI following the surgery	Death
Our case	2024	65	Female	Aortic valve replacement	Coronary angiography identified a dissection from the ostium of the LMCA to the ostium of the LCx	IVUS guidance PCI	Cardiogenic shock secondary to acute MI	Survival

The etiology of LMCA dissection following cardiac surgery involves several potential factors, such as the propagation of dissection from the aortic root, direct infusion of cardioplegia solution into the coronary artery, and intraoperative maneuvers, paticularly in cases with severe calcification of the aortic root. In the majority of documented clinical cases, the precise etiology remains uncertain (Table 1). In our case, the most plausible explanation is presumed mechanical injury to the left coronary ostium during the removal of mechanical aortic valve. This inference is supported by the challenges encountered during the extraction of the mechanical aortic valve and the subsequent development of hemodynamic instability coupled with ventricular arrhythmias immediately post-removal of the aortic clamp, indicative of exacerbation of pre-existing coronary artery dissection.

Current data do not offer a clear consensus or guideline for the treatment of LMCA dissections. In our case, the patient experienced hemodynamic instability and immediate onset of ventricular arrhythmias following aortic valve replacement surgery. While grafts were successfully established to revascularize the LAD and RCA, concerns regarding potential injury and heightened risk of myocardial rupture during the rotational manipulation required to establish a graft to the LCx in a patient with a pre-existing mechanical mitral valve led the surgical team to opt against further intervention. Following surgery, the patient continued to exhibit persistent indications of low cardiac output syndrome and ventricular arrhythmias, which remained unresponsive to treatment, prompting the consideration for coronary angiography.

In coronary artery interventions, particularly those involving LMCA, the utilization of intravascular imaging has garnered recommendations from various cardiology societies. The European Society of Cardiology advised considering the use of IVUS to optimize the outcomes of PCI for unprotected LMCA disease with class IIA level of evidence (7). Meanwhile, the role of IVUS in complex coronary interventions such as bifurcation lesions, calcified lesions, or bifurcation lesions in Asian countries is recommended with a higher level of evidence with class I (8). However, the utility of IVUS or optical conherence tomography in managing LMCA dissections following valve replacement surgery in unstable patients remains underexplored due to limited available data. This case represents the third instance of intravascular imaging implementation in such scenarios, with OCT having been employed once before, and IVUS now being documented for the second time amidst hemodynamic instability. With IVUS support, we successfully precisely evaluate the underlying mechanism and characteristics of injury, confirm the positioning of the guidewire, and enhance the efficacy of PCI.

Regarding the patient's condition post-discharge, the LVEF has not significantly improved compared to the time of hospitalization, although blood pressure and heart rate have allowed for the escalation of foundational heart failure medications. The dosages of the patient's heart failure medications have remained nearly constant over the past four months, and SGLT-2 inhibitors have not yet been initiated. This needs to be addressed promptly to improve cardiac function and reduce future cardiovascular events for the patient. Additionally, adjusting anticoagulation therapy to achieve the target INR through patient education on diet, adherence to treatment, and regular monitoring is crucial in managing patients with mechanical valves.

## Conclusion

This case signified the importance of closely monitoring patients postoperative to promtly identify and diagnose this fatal complication following cardiac valve replacement surgery. Currently, there is no established definitive management approach for this complication. The treatment should be based on the timing of diagnosis, patient's condition, physician's expertise. In our case, where dissection is identified after surgery, PCI with the help of IVUS image was safe, effective and help to reduce the necessity for further surgery. Given the current scarcity of evidence, a multidisciplinary approach should be adopted in the management of LMCA dissection, which involve both cardiac surgeons and interventional cardiologists.

## Data Availability

The original contributions presented in the study are included in the article/Supplementary Material, further inquiries can be directed to the corresponding author.
